# Decoding the Attentional Demands of Gait through EEG Gamma Band Features

**DOI:** 10.1371/journal.pone.0154136

**Published:** 2016-04-26

**Authors:** Álvaro Costa, Eduardo Iáñez, Andrés Úbeda, Enrique Hortal, Antonio J. Del-Ama, Ángel Gil-Agudo, José M. Azorín

**Affiliations:** 1 Brain-Machine Interface Systems Lab, Miguel Hernández University, Av. de la Universidad S/N, 03202 Elche, Spain; 2 Biomechanics and Technical Aids Units, Physical Medicine and Rehabilitation Department, National Hospital for Spinal Cord Injury, SESCAM, Finca de la Peraleda S/N, 45071, Toledo, Spain; University of Electronic Science and Technology of China, CHINA

## Abstract

Rehabilitation techniques are evolving focused on improving their performance in terms of duration and level of recovery. Current studies encourage the patient’s involvement in their rehabilitation. Brain-Computer Interfaces are capable of decoding the cognitive state of users to provide feedback to an external device. On this paper, cortical information obtained from the scalp is acquired with the goal of studying the cognitive mechanisms related to the users’ attention to the gait. Data from 10 healthy users and 3 incomplete Spinal Cord Injury patients are acquired during treadmill walking. During gait, users are asked to perform 4 attentional tasks. Data obtained are treated to reduce movement artifacts. Features from *δ*(1 − 4*Hz*), *θ*(4 − 8*Hz*), *α*(8 − 12*Hz*), *β*(12 − 30*Hz*), *γ*_*low*_(30 − 50*Hz*), *γ*_*high*_(50 − 90*Hz*) frequency bands are extracted and analyzed to find which ones provide more information related to attention. The selected bands are tested with 5 classifiers to distinguish between tasks. Classification results are also compared with chance levels to evaluate performance. Results show success rates of ∼67% for healthy users and ∼59% for patients. These values are obtained using features from *γ* band suggesting that the attention mechanisms are related to selective attention mechanisms, meaning that, while the attention on gait decreases the level of attention on the environment and external visual information increases. Linear Discriminant Analysis, K-Nearest Neighbors and Support Vector Machine classifiers provide the best results for all users. Results from patients are slightly lower, but significantly different, than those obtained from healthy users supporting the idea that the patients pay more attention to gait during non-attentional tasks due to the inherent difficulties they have during normal gait. This study provides evidence of the existence of classifiable cortical information related to the attention level on the gait. This fact could allow the development of a real-time system that obtains the attention level during lower limb rehabilitation. This information could be used as feedback to adapt the rehabilitation strategy.

## Introduction

According to the World Report on Disability provided by the World Health Organization (WHO), more than 1000 million people from the entire world suffer some sort of disability, which represents the 15% of the world population. Between 110 and 190 millions of adults have significant difficulties to perform daily activities. The number of disabled people is rising due to the population ageing and the increase of chronic diseases [1]. Spinal Cord Injury (SCI) is one of the most concerning diseases that lead to motor disability. According to the National Institutes of Health (NIH), among neurological disorders, the cost to society of SCI is exceeded only by the cost of mental retardation [2]. These considerable costs come from the rehabilitation devices, clinical staff and the long time periods required to perform the rehabilitation therapies needed by SCI patients. Emerging from these problems, governments are investing on technologies oriented to improve rehabilitation therapies and many research groups are focusing their studies on this topic [3–5].

In classical physical rehabilitation, patients are rehabilitated by therapists or devices that induce on them the movements they cannot do on their own. Over the last years, the field of neurorehabilitation has proved in multiple occasions that this process could be widely improved by involving the patients in a neurological way [6]. The brain is a learning organ, capable of restoring lost neural paths during the rehabilitation process. Using this capability, known as neuroplasticity [7], it is possible to reduce rehabilitation periods and improve the recovery results. To that end, it is necessary to provide some sort of neurological feedback to the patient during the rehabilitation. In [8–10], a virtual reality has been used to provide the patients with visual feedback of an avatar performing, simultaneously, the rehabilitation movements. Results show high improvements in the rehabilitation process and also in the patient’s motivation and involvement. Another way of providing neurological feedback is through the use of Brain-Computer Interfaces (BCI) [11, 12]. BCIs obtain neural information from the brain by acquiring the electrical signals on the scalp. This information can be used to obtain patient’s intentions and mental state. Using patient intentions to trigger the rehabilitation strategy enhances the effectiveness of the therapy in terms of time and performance [13]. In [14], an upper limb rehabilitation system is tested on stroke patients showing evidence that, among this type of patients, a robotic rehabilitation based on a motor imagery BCI result in greater motor improvement than standard robotic rehabilitation. On the other hand, there are BCIs designed to obtain parameters related to cognitive mechanisms like concentration, workload and attention. These parameters play an important role during rehabilitation as the mental state of the patient has a huge influence over the therapy performance [15].

In the case of lower limb rehabilitation, attention has been proved to be an important parameter that affects the final performance of the therapy [16, 17]. Slow gait and poor stability have been also associated to a low attention and cognition capabilities [18, 19]. In [20, 21], dual-task strategies are also used to test the gait stability and variability in elder people according to the attention paid on the gait process showing similar results. In BCI studies, attention has been a well studied parameter, but it is susceptible to be subjectively understood depending on the focus of each specific work. Attention and cognitive mechanisms have been related to different electroencephalographic (EEG) phenomena produced mainly on the alpha, beta and gamma bands. Power spectral variations on alpha and beta bands have been related to changes on brain’s attentional demands [22–24] and the increase of theta/beta bands power ratio and alpha band peaks have been used in [25] to diagnose attention-deficit and hyperactivity disorders. Phase synchronization on gamma band has been related to visual-spatial selective attention [26–29], which is the act of focusing on a particular object, action or stimulus for a period of time, while simultaneously ignoring irrelevant information that is also occurring. Also, amplitude changes of evoked potentials like P300 have been related to the level of attention paid to an external stimulus [30, 31].

In the current work, a evaluation study is performed to find a relationship between the attention level that a user pays to the gait and the EEG brain signals. The main goal of this study is to set up the basis for a future system capable of classifying in real time the attention level of SCI patients during lower limb rehabilitation. The development of such system could have important implications on the rehabilitation strategies, being a first step to a rehabilitation system capable of changing the therapy parameters in order to fit the mental state of the patient. To perform this study, cortical information from experiments where healthy users and incomplete SCI patients walk on a treadmill are extracted during several tasks related to the attentional demands on gait. This study faces two critical points. The first one is to evaluate the validity of the EEG signals measured during walking, which is a subject of controversy in this field. In [32] it is stated that it is possible to measure good quality EEG signals during movements like walking, cycling and sitting to measure attention by evaluating P300 amplitudes (1–4 Hz), on the other hand, a recent study [33] claims that, during treadmill walking, the EEG signals are polluted with movement artifacts that change depending on the subject, conditions, electrodes and strides, affecting mainly low frequencies (∼1 − 8 Hz) and which cannot be removed just using the current artifact removal techniques. The second point is to correlate the results obtained with the state-of-the-art studies to find a relationship between the attention paid on the gait an the current investigations on attention and cognitive mechanisms.

To deal with these problems, all frequency bands associated to cortical signals are evaluated to find those optimal to classify the attention parameters of interest and, after that, several classifiers are tested to get the one that provides better results. The experimental conditions have been defined to avoid the maximum amount of noises and the signals are processed with artifact removal algorithms in order to reduce, as much as possible, their contributions and evaluate only the cortical contribution.

## Materials and Methods

### Acquisition system

EEG data are acquired through 32 channels using pseudo-active electrodes located on the scalp through an elastic cap (g.GAMMAcap, g.Tec, GmbH, Austria) with the following spatial distribution: FZ, FC5, FC3, FC1, FCZ, FC2, FC4, FC6, C5, C3, C1, CZ, C2, C4, C6, CP5, CP3, CP1, CPZ, CP2, CP4, CP6, P5, P3, PZ, P4, P6, PO7, PO3, POZ, PO4 and PO8 according to the international system 10/10 using AFz position as ground and a monoauricular reference in the right earlobe. Electrical signals are preamplified (g.GAMMAbox, g.Tec, GmbH, Austria) before their 1200 Hz digitalization using two commercial amplifiers (g.USBamp, g.Tec, GmbH, Austria). A 50 Hz Notch filter is also applied to remove the power line interference.

### Experimental environment

**Fig 1:**
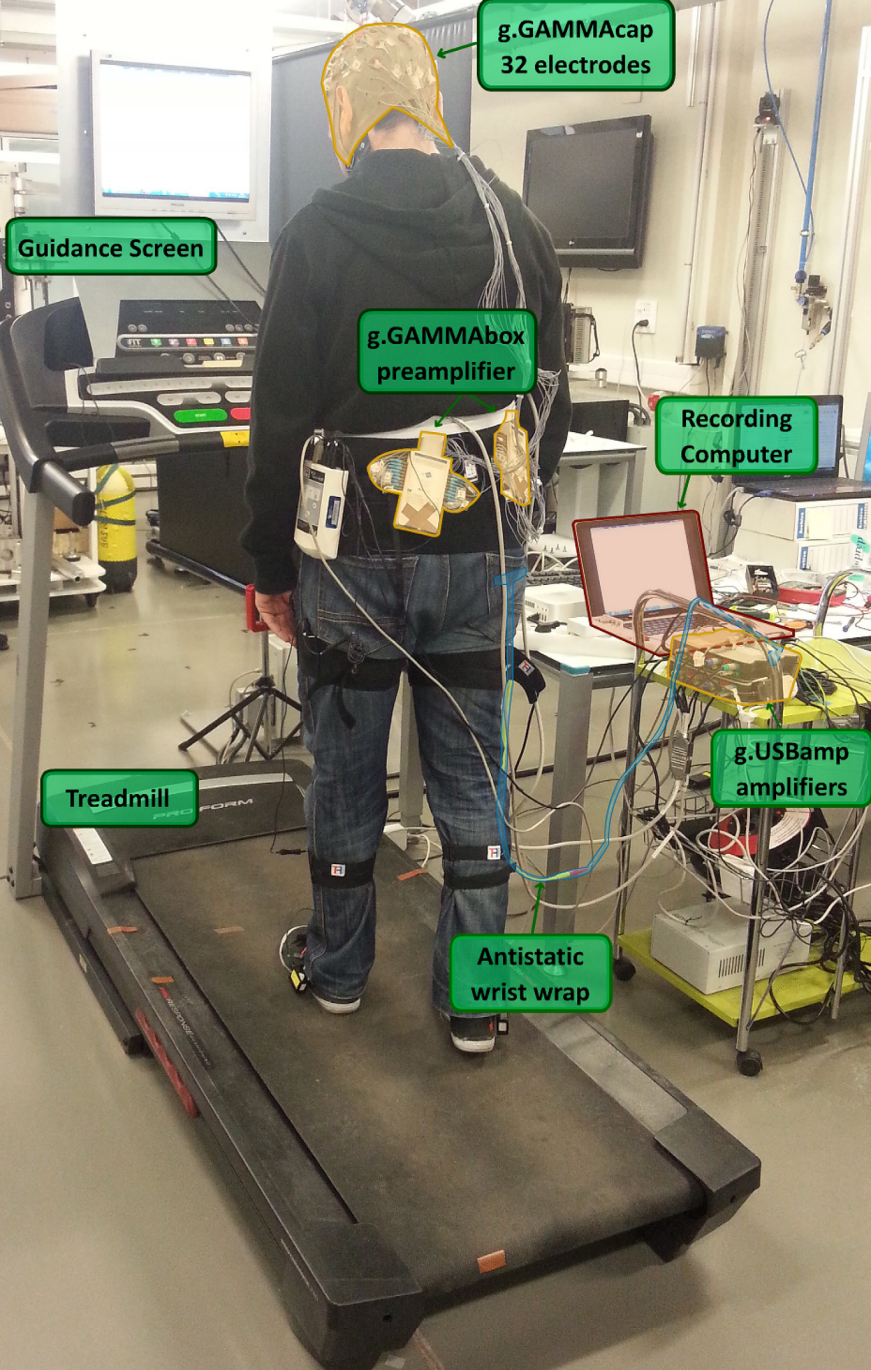
Experimental Environment. The user walks on a treadmill while a screen located at the eye-level provides guidance to perform different attention-related tasks. During the experiment, EEG signals are recorded from 32 channels located over the cortex through the g.GAMMAcap. Electrical signals are preamplified through 2 g.GAMMAboxes located in the user hip and digitalized in the g.USBamplifiers. An antistatic wrist strap connects the user’s wrist with the amplifiers ground to remove treadmill’s electrical noise. The digitalized data are recorded in a computer system.

Fig 1 shows an image of the environment of the experiments. The user is wearing the EEG cap which is connected to the amplifiers using a couple of extensor wires. A treadmill is used to create a steady gait pattern. An antistatic wrist strap connects the user’s wrist to the amplifier ground to avoid noises related to the treadmill vibration. In front of the user, a screen placed on the treadmill is connected to the recording computer to provide visual guidance to the user during the experiments.

### Experimental paradigm

**Fig 2:**
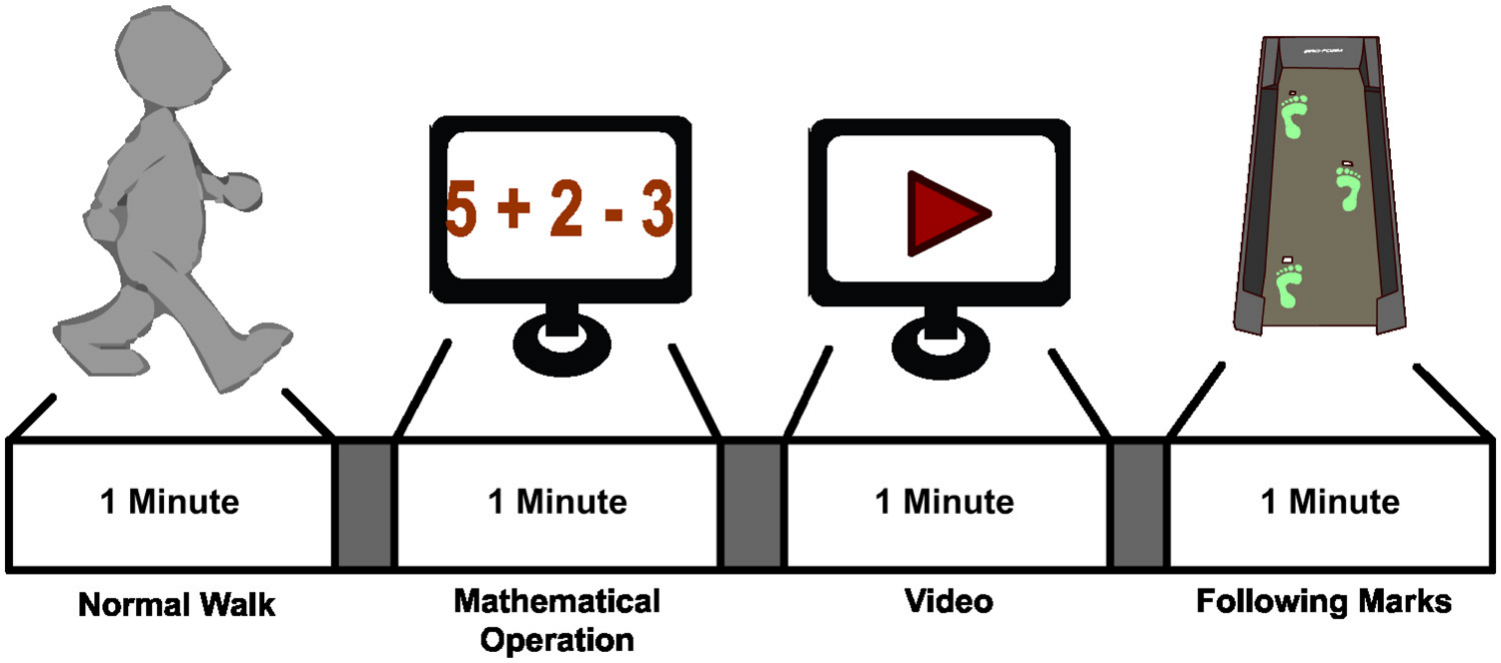
Experimental Cue. A single run is divided into 4 different tasks related to the attention level during gait: Normal walking as Standard Attention Level, performing mathematical operation and watching a video during walking as Low Attention Level, and following marks on the threadmill as High Attention Level.

The experiment performed is based on the dual task paradigm, commonly used in the literature to evaluate attention measurements [34, 35]. In this work, users walk on a treadmill at 2 km/h and 0 degrees of tilt while they are asked to perform 4 different 1-minute tasks that induce changes in the attention paid to gait. In Fig 2 a graphical representation of a run is shown. During the first task the subject walks normally, looking straight ahead, without any distraction. This task represents a standard attention level on the gait as the user is not fully focused on the gait but not distracted by any other task. In the second and the third task, the subject is asked to perform several mathematical operations and to watch a video on the screen, respectively. Mathematical operations are composed by simple additions and subtractions with numbers between 1 and 9 presented to the user in a friendly interface. The video is presented soundless with subtitles to avoid the appearance of auditory potentials and to keep the interest of the user. Both tasks represent a low attention level since the user is focusing on a non-related gait task. Finally, during the fourth task, the user is asked to walk following some marks located on the treadmill trail. These marks have been consciously located following an unsteady distribution to force the user to keep a high attention level on the variable gait pattern. The order of task performance was the same shown on Fig 2 for all sessions and subjects.

A complete session of the experiment is composed of 8 runs with 1 minute breaks between runs. The final duration of a session is approximately 40 min and it is composed of 32 min of useful data (8 minutes of each task).

### Participants

Twelve healthy users have performed 2 sessions of the experiment, 4 women and 8 men, all of them right handed, with ages between 22 and 32 (26.3 ± 3.8). Also 3 incomplete SCI patients have performed 1 session of the experiment, all of them right handed men, with ages between 26 and 58 (44 ± 16.3). Healthy users are degree and Ph.D. students from the Miguel Hernández University of Elche with no known diseases, and patients are from the National Hospital for Spinal Cord Injury (Spain) and they have incomplete SCI with motor lesions between C5 and C6 level. All patients selected were able to walk by themselves or using simple assistive devices like crutches or walkers. They do not suffer from cerebral injury so their brain processes should not present huge differences from those manifested on healthy users [36]. All users have been previously informed about the experimental procedure and they have signed an informed consent according to the Helsinki declaration. The experimental procedure were approve by the ethics committee of the Miguel Hernández University of Elche (Spain).

### Processing and features extraction

This section describes how to obtain the frequency features of the signal for all the bands where cortical information can be found. According to literature [37] these bands are: delta (*δ* = 1–4 Hz), theta *θ* (*θ* = 4–8 Hz), alpha (*α* = 8–12 Hz), beta (*β* = 12–30 Hz), low gamma (*γ*_*low*_ = 30–50 Hz) and high gamma (*γ*_*high*_ = 50–90 Hz) bands.

#### Time processing

Although cortical processes can be described and generalized, electrocortical signal’s amplitudes experience huge changes in the time domain depending on the user and the recording day [38]. Also, recording EEG signals during human walking induces several sources of noise that contaminate the cortical signals of interest [33]. For these reasons it is important to apply processing methods to remove the artifacts affecting the brain signals and also a standardization of the signals to make possible the comparison between users and sessions.

**Fig 3:**
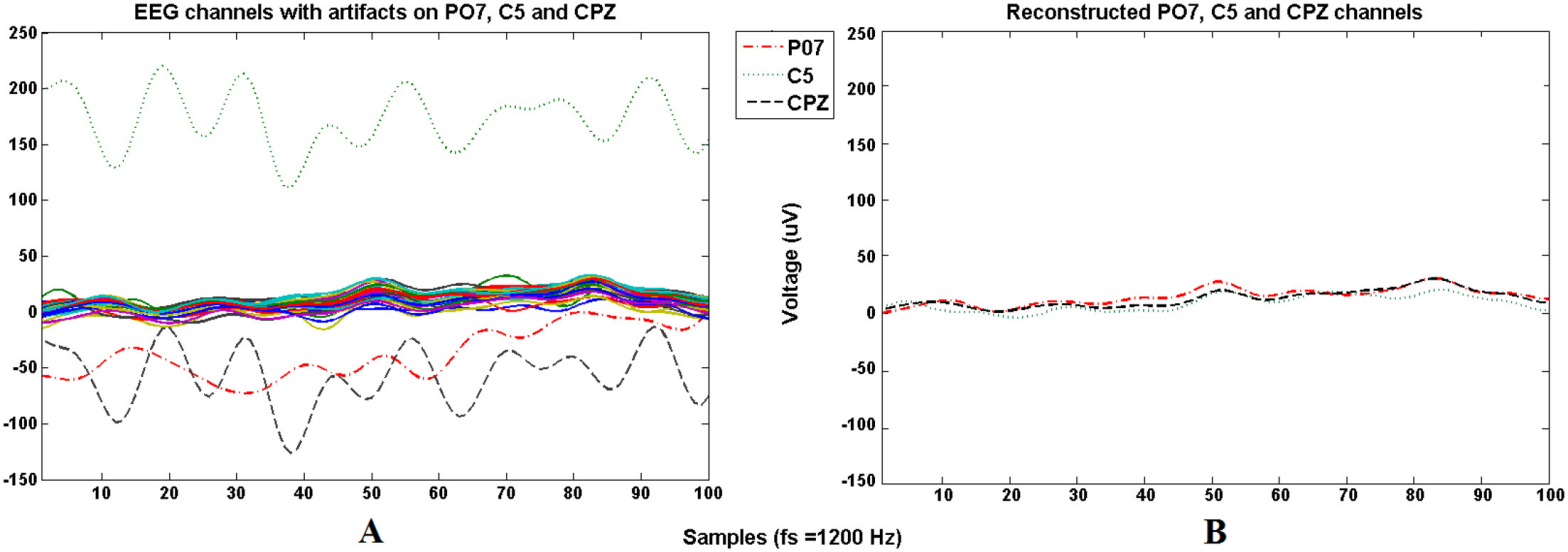
Noisy channels reconstruction. Graph A shows 100 samples of the 32 electrodes time signals from the first session of User 1. Solid lines represent the artifact-free electrodes (±50 uV, common range of EEG signals), dotted and dashed lines represent the noisy channels (PO7, C5 and CPZ, out of ±50 uV range). Each noisy channel is replaced by the average value of the surrounding channels. Graph B shows the reconstructed signal.

First, all data are bandpass filtered between 0.5 and 100 Hz to remove blink artifacts associated to low frequencies [39] and electromyographic (EMG) artifacts associated to high frequencies [40]. After that, each channel from every session is visually inspected to find outliers. On Fig 3A, the first session of user 1 is shown to graphically see the artifacts found on some channels during a recording. The simplest way to deal with these channels is their rejection with the consequent loss of information. In this work, to keep some information from these channels and preserve the data dimensionality and the final number of features extracted, the noisy channels are going to be removed and reconstructed using data from a spatially distributed set of recordings sites by means of spatial position as in [41]. Using this method no extra information is added to the signal (only redundant information from surrounding electrodes), allowing the recovery of some lost information from a specific scalp area using the information left on the neighbor areas due to volume conduction [42, 43]. Fig 3B shows the signals after replacing the contaminated areas. The amount of reconstructed information from noisy electrodes is the 2.70% of the total available data from both healthy users and patients.

The next step is the standardization of the signals in the time domain. To do that it is necessary to find a parameter that represents the brain signals and that does not experience huge variations during a session. Also, an important aspect of this process is to reduce to its minimum the loss of information after applying the standardization processes. The selected parameter to perform this standardization has been developed during this research and for simplifications it is going to be referenced as Maximum Visual Threshold (MV Threshold). This parameter is computed by windowing an EEG channel in L-samples epochs and averaging the maximum value of each epoch. Eq (1) shows the formula to obtain the MV Threshold for the electrode *e* where $X_{\left(i-1)\cdot L+1:i\cdot *L\right)}^{e}$ is the *L*-samples epoch number *i* for *i* = 1, 2, 3…*N* being *N* the total number of epochs.

$$\begin{array}{c} MVThreshold^{e}=\frac{1}{N}\sum_{i=1}^{N}max\left(X_{\left(i-1)\cdot L+1:i\cdot *L\right)}^{e}\right) \end{array}$$ (1)

**Fig 4:**
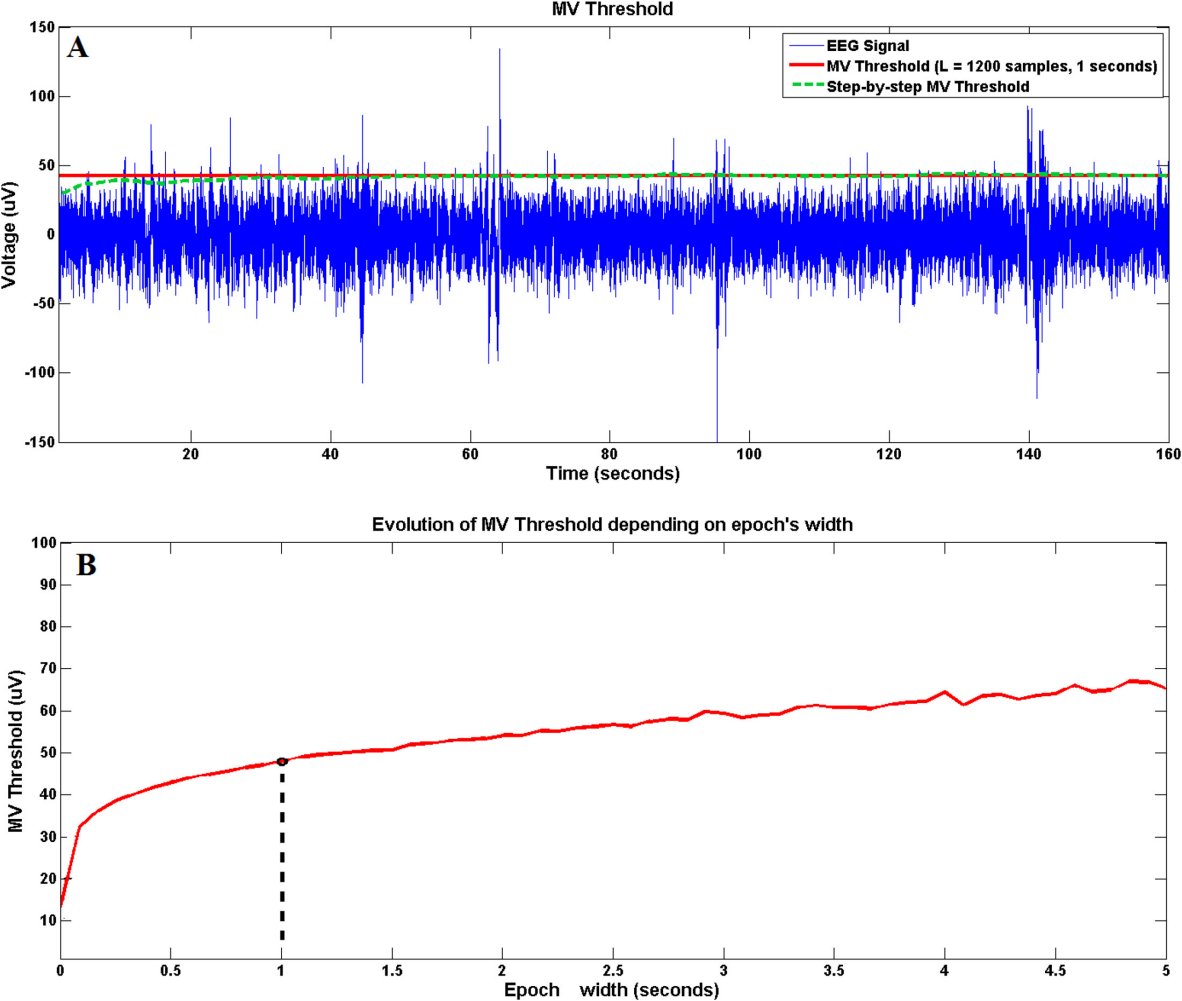
Maximum Visual Threshold. Graph A shows 160 seconds of a single channel EEG signal (in blue) and the MV Threshold computed for that signal with a epoch width (L) of 1200 samples (1 second) (in red). Graph B shows the evolution of the MV Threshold depending on the width L of the epochs for the same EEG channel. The black point shows the value selected and shown in graph A.

In Fig 4A a highpass filtered EEG signal is shown in blue. The red line represents the MV Threshold calculated for L = 1200 samples (1 second). The green dotted line represents the step-by-step computation of the MV Threshold. During the initial epochs, the average variation (green line) presents an unsteady behavior but after averaging several tens of epochs, the variation is very small and it is robust against isolated high amplitude peaks. Since the value of the MV Threshold depends on the epoch width *L*, it is important to select this *L* value taking into account the width of the epochs that are going to be used to obtain the features of our signals. Fig 4B shows the MV Threshold for different values of *L*. The black point shows the one selected for the current work. For *L* values close to 1 (0.83 milliseconds), the value of MV Threshold is really close to the signal average which is almost 0. Prior to *L* = 110 (92 milliseconds) the value of MV Threshold presents an accelerated increase and it becomes more stable after this point. This phenomenon is closely related to the amount of spectral information of the signal. The minimum epoch length needed to get all the spectral information desired from a signal is computed as *t*_*min*_ = 1/*f*_*min*_, being *f*_*min*_ the minimum frequency of interest in the signals analyzed. In this case, the *L* = 110 is associated to low frequencies (∼10 Hz) corresponding to the *α* band, the activation of which is expected from EEG data obtained during motor tasks, implying that most of signal information appears in this band. However, current EEG data are bandpass filtered between 1–100 Hz which means that the minimum epoch length to have access to all the bandwidth is 1 seconds (*L* = 1200) which also in the range where MV Threshold presents a stable behaviour.

The MV Threshold is calculated for each electrode and it can be considered constant during a whole session. For each session, 32 MV Thresholds are computed, one per channel with L = 1200 samples. The signal standardization is computed according to Eq (2) where *V*(*t*)_*e*_ is the time domain EEG signal of the electrode *e* and *MVThreshold*_*j*_ is the MV Threshold of the channel *j*, with *j* = 1, 2, 3, …*Ch* being *Ch* the total number of channels.

$$\begin{array}{c} SV{\left(t\right)}_{e}=\frac{V{\left(t\right)}_{e}}{\frac{1}{Ch}\cdot \sum_{j=1}^{Ch}\cdot MVThreshold_{j}} \end{array}$$ (2)

The values of the MV Thresholds are different on each session and user, but they remain constant during a single session. The use of this standardization makes possible the comparison of data between users without the loss of information. The bandwidth of interest can be recovered from the signals and the variation of spectral power between electrodes is not lost by averaging the MV Thresholds obtained for each of the channels.

#### Features extraction

Once the signals are filtered and standardized, breaks from each session are removed and the remaining data is divided into one minute tasks (4 per run). This division is performed prior to epoch segmentation to avoid the existence of epoch containing cortical information of two consecutive tasks. Each task is divided into 1 second epochs (1200 samples) with an overlap of 0.5 seconds (600 samples) between epochs obtaining 3840 epoch per session ($\frac{8\;Runs\;\cdot \;4\;Tasks\;\cdot \;60\;Seconds}{0.5\;Seconds\;(Overlap)}$) and 960 epochs per task (4 attention tasks). Epoch and overlap values are selected to fit future real time specifications and they are also useful for the analysis proposed in this work related to frequency bands comparison and task classification. After that, the spectrum of every epoch is computed using the Maximum Entropy Method presented by Burg in [44]. This method is an autoregressive calculation technique introduced to compute the spectrum finite-length epochs of real time sampled data. The AR-parameters are computed by minimizing the sum of the square forward and backward prediction errors [45]. This method reduces the minimum entropy components of the signal which are associated to those components than provide less amount of information reducing this way the random components (usually associated with electronic noise) and improving the components with more information. For each epoch the spectrum is computed between 1 and 100 Hz with a spectral resolution of 1 Hz.

The computed spectrum of the epochs is used to obtain 6 different features sets according to the frequency bands analyzed. As it was mentioned at the beginning of the processing section, the frequency bands evaluated are delta *δ* (1–4 Hz), theta *θ* (4–8 Hz), alpha *α* (8–12 Hz), beta *β* (12–30 Hz), low gamma *γ*_*low*_ (30–50 Hz) and high gamma *γ*_*high*_ (50–90 Hz). For each frequency band and epoch, 32 features are obtained as the sum of all frequencies between the lowest and the highest frequency of each band (for each channel).

This feature extraction process is performed for each individual session, i.e twice per each healthy subject and once per each patient.

#### Frequency band selection

To take an objective decision regarding the optimal frequency band to classify the attention on the gait, there are several possibilities based on the use of mathematical parameters and indices that provide information related to class differentiation. In [46], Millán et al. introduce a coefficient that expresses the separability between 2 classes according to their mean and standard deviation across all the epochs. To use this coefficient it is necessary to obtain a single value of mean and standard deviation per class which is difficult when each task is represented by more than one feature per epoch. A more suitable parameter for the present study is the Battacharyya distance (*Bdist*) which was firstly introduced by Battacharyya in [47]. This parameter provides a measurement of the similarity of 2 sets of features and it is closely related to the Battacharyya coefficient which is a measure of the amount of overlap between 2 statistical samples or populations. According to literature, this measurement has widely proved its reliability for signal selection purposes [48–50]. As mentioned, *Bdist* is used to compare 2 classes, however in the current work, to obtain information related with the separability of 4 classes, the *Bdist* has been calculated for all the possible combinations of the 4 classes.

Since the *Bdist* can provide any value in the range 0 < *Bdist* < ∞, it is necessary to use a selection criteria to decide which frequency bands present the higher class separability. In [51], Choi and Lee have performed a study where they represent the Bayes error [52] of 2 task classification against the Battacharyya distance between 2 classes. As a result, they obtain a logarithmic behavior being the Bayes error between ∼10% and 30% for *Bdist* = 0.5, ∼1% and 7% for *Bdist* = 2 and ∼0% and 2% for *Bdist* = 3.5. For this work, 2 classes are going to be selected as highly separable if their *Bdist* = > 3.5. Only features from healthy subjects are used in the computation of *Bdist* values. They represent most of the cortical data recorded during this work and the attention level paid from patients is expected to be higher during non-attentional tasks. Best features selected by this method are applied to patients data during the classification stage.

### Classification

Five different classifiers have been considered to test the capability of distinguishing between the 4 gait attention tasks. The classifiers used are properly enumerated below:

1. Support Vector Machine (SVM) [53] with Radial Base Function kernel with *C* = 512 and *y* = 0.002 (parameters obtained in a previous work [54]).
2. Naïve Bayes (NB)[55].
3. Linear Discriminant Analysis (LDA) which is a generalization of Fisher’s linear discriminant [56].
4. K-Nearest Neighbors (KNN) [57] with the number of neighbors *k* = 30.
5. Decision Tree Learning (DTL) [58].

To validate the success rate results obtained by each classifier for each session (2 session x 12 healthy users + 1 session x 3 patients = 27 sessions) an 8-fold cross validation has been used where each run has been used as fold.

### Chance level computation

To validate the results and to select the best classifier, it is necessary to confirm the significance level between the classification results and the chance level. Applying the simplest mathematical statement, the chance level for a 4-task classification system assuming class equality (which is the case of the current study) is 25%. But for real finite data analysis the chance level presents several variations according to the specific conditions of the data studied. In [59], Müller-Putz et al. introduce a mathematical method to calculate the range of values corresponding to the chance level according to the number of tasks classified and the number of epochs used in the classification stage for a 2-tasks classification system. According to Müller-Putz’s work, the confidence interval around the expected chance level can be calculated using Eq (3) where *n* is the number of epochs, $z_{1-\frac{\alpha }{2}}$ is the $1-\frac{\alpha }{2}$ quantile of the standard normal distribution *N*(0, 1), *α* is the level of confidence required (typically 0.1 and 0.05) and $\tilde{p}$ is the unbiased estimator computed according to Eq (4). In this case, *n* is still the number of epochs and $\bar{X}$ is the averaged probability of all the individual probabilities of a correct classified epoch *X*_*i*_ with a classifier that performs a random classification. The Eq (5) shows the mathematical formula to compute $\bar{X}$, which according to the definition provided corresponds to the mathematical value of the chance level for an ideal random classifier (50% for 2 tasks). For more than 2 classes the model can be extended as shown in [60].

$$\begin{array}{c} Chance\;Level\;Range=\tilde{p}\pm \sqrt{\frac{\tilde{p}\cdot \left(1-\tilde{p}\right))}{n+4}}\cdot z_{1-\frac{\alpha }{2}} \end{array}$$ (3)

$$\begin{array}{c} \tilde{p}=\frac{n\cdot \bar{X}+2}{n+4} \end{array}$$ (4)

$$\begin{array}{c} \bar{X}=\frac{1}{n}\cdot \sum_{i=1}^{n}\cdot X_{i} \end{array}$$ (5)

## Results

### Features spatial representation

**Fig 5:**
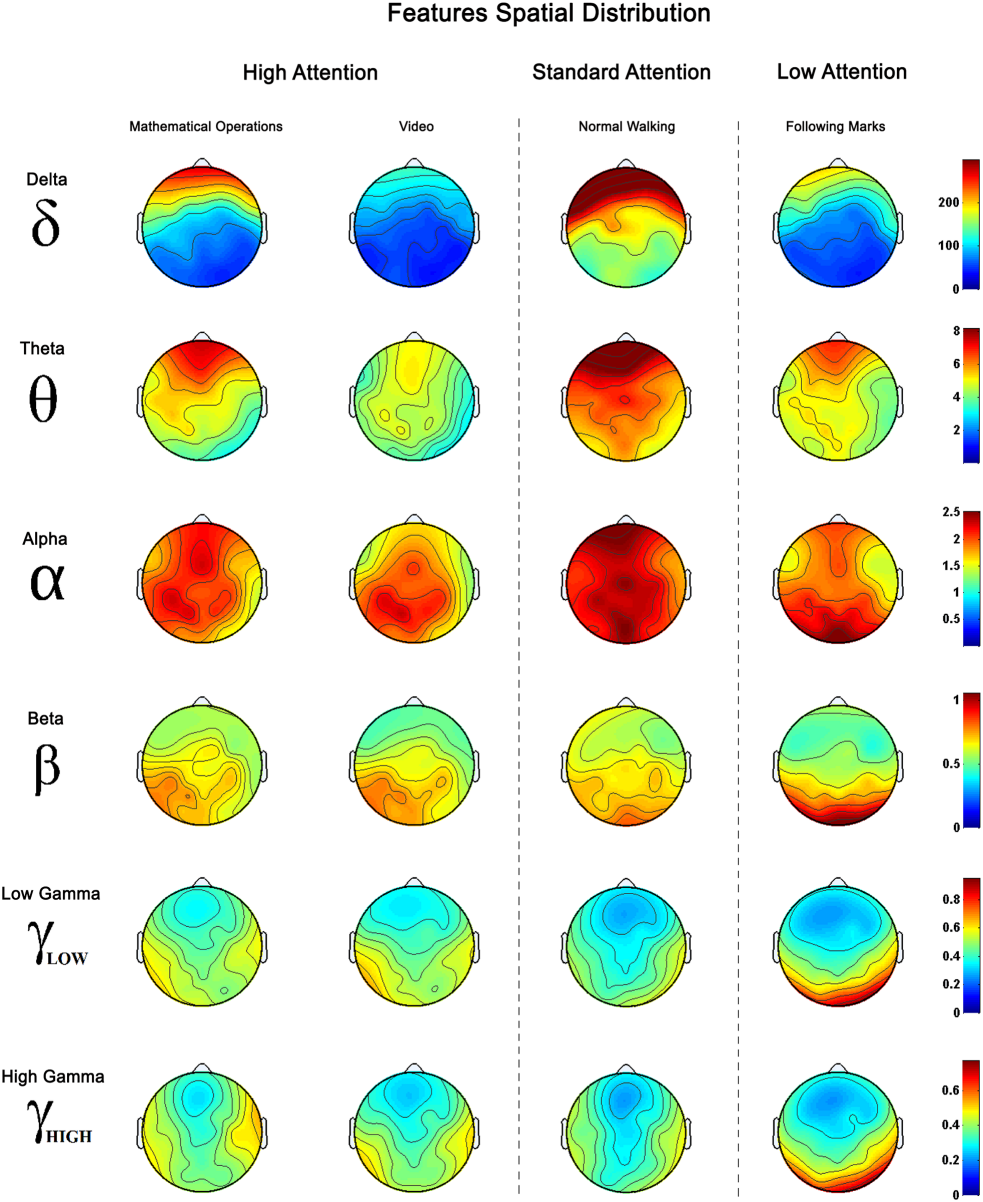
Features Spatial Distribution. Spatial distribution is represented for each task and frequency. Features used are computed by averaging the features of every subject (healthy users and patients) and sessions for each task and frequency band. Tasks are arranged according to the increasing attentional demand.

After the signal epoch segmentation and features extraction, a single 32 features vector is computed for each frequency band and task as the average of the epochs of all sessions (from both healthy and patients data). This makes 6 frequency bands per 4 tasks, a total of 24 feature vectors. The spatial distribution of these features has been represented in Fig 5 in order to visually appreciate the difference between each task depending on the frequency range evaluated. On this representation it is possible to appreciate some differentiation between tasks and frequencies. Focusing on the minimum and maximum spectral power values used to represent each frequency, it is shown that the spectral power of EEG signals gets lower when the frequency evaluated increases. These results, based on visual inspection and spectral power level, are quite subjective in order to decide which frequency bands provides more information to differentiate between the attention tasks presented.

### Frequency band separability

To obtain an objective measurement related to class differentiation, the *Bdist* is used. In Table 1 this parameter is shown for each 2-tasks combination and frequency band. The letter labelling for the class combination is the following one: *A* for “Normal Walking” class, *B* for “Mathematical Operations” class, *C* for “Video” class and *D* for “Following Marks” class. All the tasks combinations that achieve the criteria selected (*Bdist* = > 3.5) have been highlighted in bold in Table 1. Also the first and second maximum values for each combination are marked with one or two asterisks respectively.

**Table 1:** Bhattacharyya distance. Values of *bdist* for the paired combination of tasks on each frequency band. All *bdist* values > 3 are marked in bold. Highest *bdist* values for each task combinations are marked with * and second highest *bdist* values are marked with **.

	AB	AC	AD	BC	BD	CD
**1–4Hz**	16.62*	17.86*	16.32*	1.68	1.52	1.68
**4–8Hz**	**9.25**	**9.76**	**9.63**	0.85	1.36	1.20
**8–12Hz**	**8.68**	**9.32**	**9.35**	1.06	1.84	1.60
**12–30Hz**	**8.82**	**9.32**	**9.89**	1.35	2.95	2.61
**30–50Hz**	**8.22**	**8.88**	**9.64**	1.86**	4.96**	4.19**
**50–90Hz**	9.41**	10.81**	11.40**	3.98*	7.23*	5.91*

Table 1 shows high *Bdist* values for all task combinations that include task A (Normal Walking) which means that it presents a huge separability from the rest of tasks on every frequency band. On the other hand, the remaining combinations present a low *Bdist* for low frequencies achieving the selection criteria only on *γ*_*low*_ band (except for BC combination) and *γ*_*high*_ band. Another interesting fact obtained from this table is that, for all frequencies, the combination BC (“Mathematical Operation” class and “Watching Video” class) has the lowest separability features. Taking into account that these tasks or classes have been defined as the same level of attention (low gait attention level), these results suggest that, indeed, both tasks present lots of similarities. For that reason, both *γ*_*low*_ and *γ*_*high*_ bands are going to be selected for the classification stage where several classifiers are going to be evaluated in the classification of the 4 tasks mentioned.

### Classification results

The 5 classifiers previously described are applied to on the data. The average and the standard deviation for each user and classifier after an 8-fold cross validation are computed for *γ*_*low*_ and *γ*_*high*_ bands and represented on Table 2. For patients, 1 session is used to compute the success rate while results from 2 sessions are averaged in the case of healthy users. The total averaged values for each classifier and frequency band are also computed. These values are computed using all the single fold values obtained from every subject. From these results it is possible to approximate averaged classification results, e.g from high gamma band frequency features and KNN classifier, a 66.79% and a 59.08% of success rate are obtained from healthy subjects and patients respectively.

**Table 2:** Success Rates and Standard Deviation. Success rates for all subjects (10 healthy and 3 patients) and classifiers for both frequency band features.

		*γ*_*low*_				
		**SVM**	**NB**	**LDA**	**KNN**	**DTL**
**Users**	**H1**	77.38±12.38	52.02±12.37	78.10±5.66	73.38±14.03	73.29±6.49
	**H2**	68.86±12.05	44.17±9.60	67.74±9.54	65.99±13.27	59.43±13.36
	**H3**	76.89±6.45	53.52±7.43	72.99±7.38	73.71±9.96	69.76±7.13
	**H4**	68.38±3.99	36.44±3.82	62.74±4.16	66.18±3.59	61.25±4.71
	**H5**	76.37±7.62	60.62±8.01	72.31±8.05	72.73±8.54	66.32±5.81
	**H6**	70.31±15.06	45.79±8.94	69.62±14.81	67.40±14.44	61.31±9.92
	**H7**	59.36±6.47	33.81±6.73	54.52±6.43	58.05±9.09	52.07±6.63
	**H8**	68.74±7.79	37.09±6.28	59.40±5.13	66.60±8.71	60.29±6.77
	**H9**	67.00±6.85	39.22±9.82	63.37±6.16	65.31±8.36	58.94±4.69
	**H10**	57.42±6.57	31.38±7.49	54.87±5.67	52.31±9.84	50.81±5.08
	**H11**	60.51±8.24	47.41±8.93	75.38±9.71	67.24±6.69	62.39±6.86
	**H12**	71.66±9.22	61.37±5.09	72.31±6.65	72.63±7.90	68.00±5.92
	**P1**	53.66±7.00	40.95±4.33	62.72±3.92	54.09±3.94	51.83±6.47
	**P2**	67.56±4.33	37.28±5.65	62.39±10.71	71.12±5.40	63.36±4.42
	**P3**	48.28±2.96	28.99±4.16	50.54±4.18	52.05±6.54	49.14±5.15
	**Avg**	**66.16±11.54**	**43.34±12.02**	**65.27±10.53**	**65.27±11.84**	**60.55±9.95**
		*γ*_*high*_				
	**H1**	77.15±13.33	36.31±11.68	78.47±7.49	74.03±14.94	73.35±7.55
	**H2**	69.89±14.41	41.48±8.54	71.53±12.24	67.79±14.63	62.97±11.89
	**H3**	77.15±7.15	47.11±6.30	74.51±7.22	72.99±10.63	69.79±7.79
	**H4**	68.99±3.20	26.83±2.98	63.80±4.34	67.79±4.25	62.83±4.19
	**H5**	77.65±7.88	62.36±8.19	78.32±7.60	73.31±9.61	68.75±7.78
	**H6**	71.49±16.78	50.18±10.00	71.17±14.90	67.78±14.64	60.83±12.74
	**H7**	59.87±8.66	33.39±6.06	57.39±6.44	56.37±10.95	52.17±8.54
	**H8**	73.88±10.11	33.46±6.57	66.18±10.10	72.32±12.57	67.92±9.48
	**H9**	65.80±7.84	38.87±7.00	63.51±6.74	62.89±8.56	57.00±6.90
	**H10**	58.48±5.70	32.69±5.83	55.66±5.94	56.96±8.24	53.74±9.91
	**H11**	63.63±9.86	60.08±10.63	67.30±8.67	66.43±10.38	61.48±7.91
	**H12**	71.07±10.81	60.67±5.62	75.86±7.47	78.45±10.88	74.46±6.04
	**P1**	50.75±5.27	36.75±5.65	59.81±4.39	58.19±6.96	53.77±6.59
	**P2**	69.94±6.85	39.55±8.42	67.46±9.34	73.81±6.38	68.00±7.02
	**P3**	48.92±2.47	28.34±3.98	53.45±5.89	55.50±6.96	54.20±4.94
	**Avg**	**66.98±12.46**	**41.87±11.95**	**66.96±11.59**	**66.97±12.52**	**62.75±10.82**

#### Significance against chance level

**Fig 6:**
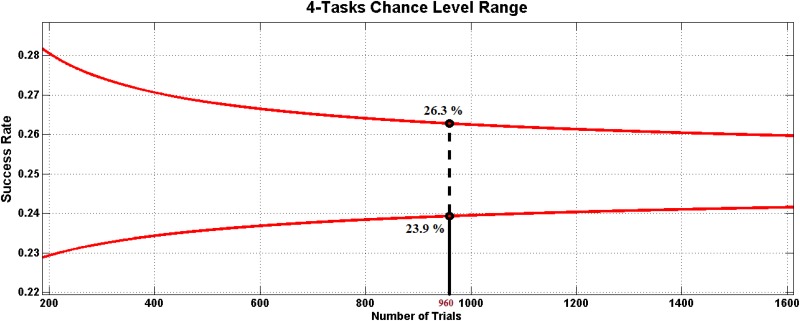
Chance Level Range. The range of variation of the chance level for a 4-task classification system is shown depending on the number of epochs classified. The top and bottom lines represent the highest and the minimum values admissible to consider the current classification random. These values are selected for the number of epochs of the current work (*n* = 960).

**Fig 7:**
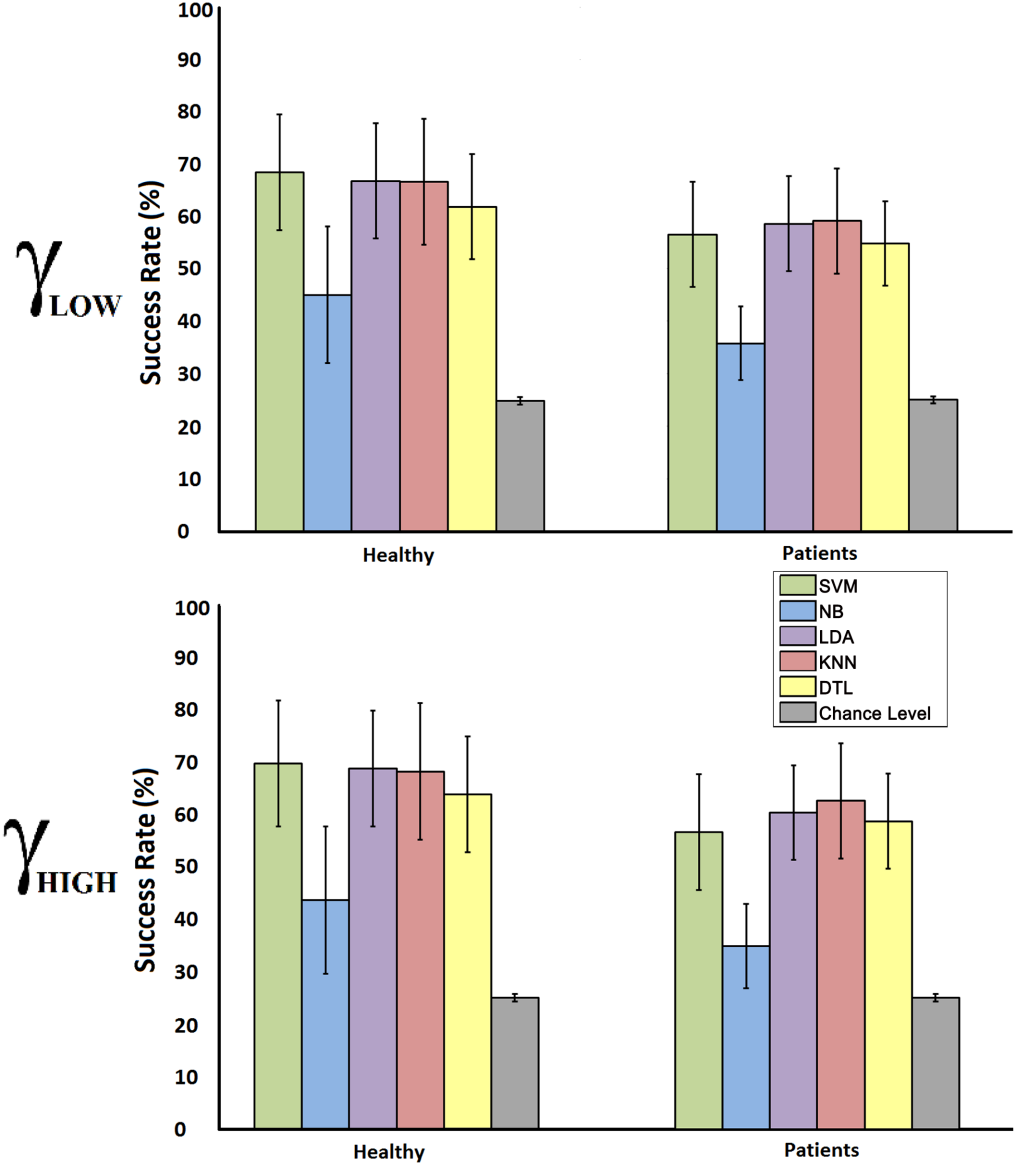
Chance Level Range. The graphs show the average success rate of each classifier and the computed chance level for 4 equally distributed tasks and the amount of epochs used during the cross validation. Graph A shows the results for *γ*_*high*_ features, while graph B shows the results for *γ*_*low*_ features.

In the current work, for each session there are 3840 epochs but only 960 are used to test the classification results on each fold iteration. The chance level range of a single fold (from the 8-cross fold validation) is computed for *n* = 960, $\bar{X}=0.25$ (4 tasks) and *α* = 0.05 (according to Eq 3), obtaining the range 23.9 < *successrate* < 26.3 (Fig 6). Any iteration of the 8-cross fold validation providing a classification result within this range should be considered random. Fig 7 shows the average success rate of each classifier for healthy users and patients on both frequency bands (24 *sessions* ⋅ 8 *folds* = 192 *success* *rate* *values* of healthy users and 3 *sessions* ⋅ 8 *folds* = 24 *success* *rate* *values* *for* *patients*) and the computed value of the chance level for each group (a 192/24 vector, respectively, with random values from the chance level range computed). The significance of these success rates is analyzed from these results. To that end, the chance level is compared to the success rate obtained for each classifier running a Wilcoxon Sum-Rank Test with a confidence interval of 95% and then applying a Bonferroni correction for multiple comparisons. This analysis shows that the success rates are predominantly above chance levels (*p* < 0.05) for all 5 classifiers.

#### Significance between classifiers, features sets and subjects

To test the significance between classifier outputs, feature sets and subjects, the same statistical approach is applied. Table 3 shows the significance *p* values of comparing the same classifier for 4 different conditions: 1) *γ*_*low*_ features: healthy against patients; 2) *γ*_*high*_ features: healthy against patients; 3) Healthy: *γ*_*low*_ against *γ*_*high*_ features; 4) Patients: *γ*_*low*_ against *γ*_*high*_ features. Table 4, on the other hand, shows the significance *p* values for all the classifier combinations for the 4 graphs of Fig 7. On both tables, the significant *p* values are marked in bold after applying a Bonferroni correction.

**Table 3:** Significance between classifiers. Significance values for the paired combination of classifiers for healthy and patient both using *γ*_*low*_ and *γ*_*high*_ features.

	**Healthy**_**γ**_**low**__			
	SVM	NB	LDA	KNN
NB	**2.2 ⋅ 10**^**−40**^	-	-	-
LDA	**2.0 ⋅ 10**^**−3**^	**5.4 ⋅ 10**^**−36**^	-	-
KNN	2.6 ⋅ 10^−2^	**4.1 ⋅ 10**^**−35**^	3.5 ⋅ 10^−1^	-
DLT	**6.2 ⋅ 10**^**−11**^	**1.5 ⋅ 10**^**−29**^	**4.9 ⋅ 10**^**−4**^	**8.0 ⋅ 10**^**−6**^
	**Healthy**_**γ**_**high**__			
NB	**2.7 ⋅ 10**^**−8**^	-	-	-
LDA	5.8 ⋅ 10^−2^	**2.0 ⋅ 10**^**−41**^	-	-
KNN	4.3 ⋅ 10^−2^	**4.1 ⋅ 10**^**−38**^	8.5 ⋅ 10^−1^	-
DLT	**8.8 ⋅ 10**^**−5**^	**6.4 ⋅ 10**^**−35**^	**1.0 ⋅ 10**^**−4**^	**1.7 ⋅ 10**^**−4**^
	**Patient**_**γ**_**low**__			
NB	**2.7 ⋅ 10**^**−8**^	-	-	-
LDA	5.1 ⋅ 10^−1^	**7.6 ⋅ 10**^**−9**^	-	-
KNN	2.7 ⋅ 10^−1^	**5.6 ⋅ 10**^**−9**^	9.8 ⋅ 10^−1^	-
DLT	5.0 ⋅ 10^−1^	2.5 ⋅ 10^−8^	2.0 ⋅ 10^−1^	1.8 ⋅ 10^−1^
	**Patient**_**γ**_**high**__			
NB	**4.3 ⋅ 10**^**−14**^	-	-	-
LDA	**3.1 ⋅ 10**^**−5**^	**3.1 ⋅ 10**^**−10**^	-	-
KNN	**2.1 ⋅ 10**^**−3**^	**8.8 ⋅ 10**^**−11**^	**2.0 ⋅ 10**^**−2**^	-
DLT	**4.8 ⋅ 10**^**−6**^	**2.3 ⋅ 10**^**−9**^	**1.4 ⋅ 10**^**−4**^	**2.5 ⋅ 10**^**−4**^

**Table 4:** Significance between bands and users. Rows 1 and 2: Significance values for both frequency features between healthy and patients. Rows 3 and 4: Significance values for both healthy and patients between frequency bands.

	SVM-SVM	NB-NB	LDA-LDA	KNN-KNN	DLT-DLT
***γ*_*low*_: *Healthy*−*Patient***	**1.44 ⋅ 10**^**−6**^	**2.90 ⋅ 10**^**−3**^	**2.60 ⋅ 10**^**−3**^	**3.00 ⋅ 10**^**−3**^	**1.50 ⋅ 10**^**−3**^
***γ*_*high*_: *Healthy*−*Patient***	**1.64 ⋅ 10**^**−6**^	7.17 ⋅ 10^−2^	**6.01 ⋅ 10**^**−4**^	**3.30 ⋅ 10**^**−2**^	**2.49 ⋅ 10**^**−2**^
***Healthy*: *γ*_*low*_ − *γ*_*high*_**	2.84 ⋅ 10^−1^	**1.87 ⋅ 10**^**−2**^	**3.46 ⋅ 10**^**−2**^	3.19 ⋅ 10^−1^	7.98 ⋅ 10^−2^
***Patient*: *γ*_*low*_ − *γ*_*high*_**	9.51 ⋅ 10^−1^	5.29 ⋅ 10^−1^	6.13 ⋅ 10^−1^	2.56 ⋅ 10^−1^	1.73 ⋅ 10^−1^

## Discussion

On the spatial distribution of the averaged features shown in Fig 5 it is possible to appreciate some differentiation between tasks and frequencies. Focusing on the minimum and maximum spectral power values used to represent each frequency, it is shown that the spectral power of EEG signals gets lower when the frequency evaluated increases. In *δ* and *θ* bands, the distributions present huge deviations between tasks and the main activation is located on the frontal lobe usually associated to blinks, head movements and other physiological artifacts [61]. These results fit the statements of Kline et al. in [33] claiming that motion artifacts have higher contributions on lower frequency bands and present huge variations depending on the subject and the walking speed. *α* band shows a main activation on the motor area which is an expected behavior during walking. According to literature, the motor cortex side activated depends on the real or imagined motor movement performed [62, 63]. For that reason, during walking, left and right areas of motor cortex are alternatively activated disguising any changes related with the attention. In the *β* band, the spectral power is softer in the motor area and experiences a smooth increase in the occipital area. Finally, for *γ*_*low*_ and *γ*_*high*_ bands there is a significant reduction on the motor cortex where the power and distribution seems to be related to the attention level classified. Compared with the motor cortex, the occipital area is much more active suggesting that visual information is being processed. These results and those provided in Table 1 about class separability suggest that the measurement of the attention level on the gait is closely related to the selective attention. As it was stated at the beginning of the paper, selective attention is described as the attention paid to an external object, action or stimulus while another object, action or stimulus, simultaneously happening, is ignored. In this case, the gait process and the environmental visual information are the event and action, respectively, confronted, and the gait attention changes depending on which event is more or less ignored or attended.

Classification values on Fig 7 and the test performed show that all classifiers tested provide significant values in the classification of this attention level, confirming the existence of discernible information related to the attention level on the gait obtained from *γ* band features. On rows 1 and 2 of Table 4, each classifier from healthy users is compared with its analog on patients showing, in most cases, significant differences between them. These results imply that the classification results from patients are significantly different than those obtained from healthy. This significance could be a consequence of the inherent increased attention on gait presented on incomplete SCI patients who are always more focused on the gait than healthy subjects regardless the distraction event presented to them [18, 19]. Both healthy subjects and patients performed the same tasks and the classification model was trained according to them, but patients’ attention on gait was always higher than healthy’s during standard and low attention tasks. On the other hand, rows 3 and 4 for Table 4 show the same classifier comparison but this time between frequency bands, showing no significant values except for classifiers C2 (NB) and C3 (LDA) on healthy users. These values suggest that the information is similar in both bands and their combination in the feature extraction stage could improve the final classification results.

Finally, on Table 3, for each subject group (healthy and patients) and frecuency band (*γ*_*low*_ and *γ*_*high*_), the classifiers are compared between them showing in most cases no significant results between them. In that case, the optimal classifiers selection could be performed by the maximum average success rate. Focusing on Fig 7, classifiers SVM, LDA and KNN provide, in general the best results, being SVM performance slightly higher on healthy users and LDA and KNN on patients.

## Conclusion

In this paper, different frequency bands extracted from data of 10 healthy users and 3 incomplete SCI patients have been evaluated to find a relationship between cortical signals and the cognitive mechanisms related to the attenton during gait. The study emphasizes the difficulties to decode cortical information on low frequency (*δ* and *θ*) bands due to the artifacts generated by movement and also to classify the cortical information on *α* band related to attention which is covered up by the motor rhythms produced during gait on cortical signals. On the other hand, information related to selective attention mechanisms that relate the attention to the gait and the attention to the external environment have been found in the *γ* band. The band selection has been performed by computing a separability index between tasks using the Bhattacharyya distance and the features extracted have been tested using 5 classifiers with an 8-fold cross validation. Final results provide an average value of 67% of success rate classification for 4 attention tasks. Success rate values have been compared to chance level, showing clear significance. Healthy users have obtained significantly higher classification results suggesting that there was less class separability on patient’s which is related to the higher level of attention they pay on non-attentional tasks due to their motor disabilities.

This work settles a basis for gait attention classification. For future works this offline analysis should be performed with a higher patient population. In addition, the features extraction should be improved combining both low and high *γ* bands and using mathematical algorithms for feature selection and reduction. The number of tasks used to train the model should be reduced to 3, removing one of the high gait attention tasks and improving this way the final success rate. Current processing algorithms should be changed to fit real time condition in order to perform online tests with healthy users and patients. Also the the reference electrode standardization technique (REST) [64] should be tested on the data to improve the quality of EEG signals in terms of spatial resolution and artifacts’ identification. And, as a final goal, an online system that provides the attention level of an incomplete SCI patient under exoskeleton rehabilitation could be used to change, in real time, the parameters of the rehabilitation to fit better to the cognitive state of the patients and improve the rehabilitation performance by involving them in a deeper level.
